# Comment on ‘Oncological and Survival Endpoints in Cancer Cachexia Clinical Trials: Systematic Review 6 of the Cachexia Endpoint Series’ by Dajani et al.—The Authors' Reply

**DOI:** 10.1002/jcsm.70069

**Published:** 2025-09-24

**Authors:** Jann Arends, Olav Dajani, Iain Philips, Richard J. E. Skipworth, Tora S. Solheim, Barry J. A. Laird

**Affiliations:** ^1^ Department of Medicine I, Medical Center—University of Freiburg, Faculty of Medicine University of Freiburg Freiburg Germany; ^2^ Regional Advisory Unit for Palliative Care, Department of Oncology Oslo University Hospital Oslo Norway; ^3^ European Palliative Care Research Centre (PRC), Department of Oncology Oslo University Hospital Oslo Norway; ^4^ Institute of Clinical Medicine University of Oslo Oslo Norway; ^5^ Edinburgh Cancer Research Centre, Institute of Genetics and Cancer University of Edinburgh Edinburgh UK; ^6^ Clinical Surgery University of Edinburgh Royal Infirmary of Edinburgh Edinburgh UK; ^7^ Department of Clinical and Molecular Medicine, Faculty of Medicine and Health Sciences NTNU—Norwegian University of Science and Technology Trondheim Norway; ^8^ Cancer Clinic St Olavs Hospital—Trondheim University Hospital Trondheim Norway; ^9^ Oslo University Sykehus, European Palliative Care Research Centre, Division for Cancer Medicine, Institute of Cancer Medicine, Medical Faculty University of Oslo Oslo Norway

**Keywords:** adverse events, cachexia, cancer, clinical trials, survival

Cancer cachexia may be interpreted as a battle of an organism against a chronic attack on its integrity, resulting in a complex and potentially oscillating evolution of pathophysiological and clinical signs and symptoms. The apparent heterogeneity and variability of this syndrome so far have impeded the development of effective interventions and even precluded an agreement on the most important targets to monitor. The cachexia endpoint series was initiated to screen and search the evidence accumulated in randomized controlled anti‐cachexia trials aiming to compare the type and value of different outcome parameters [1, 2, 3, 4, 5, 6].

The analysis of oncological endpoints yielded a large heterogeneity in target populations, interventions and trial designs [2]. This, a prevalent lack of statistical rigour and the rarity of positive findings, prevented valid comparisons of interventions and endpoints. Importantly, only two out of 57 trials reported significant effects on a primary oncological outcome [7, 8], and due to statistical issues, both results need to be interpreted as exploratory.

We are thankful for the comments added to our findings by Kumar et al., which excellently amend our suggestions for the design of future anti‐cachexia trials [9].

The deleterious effects of weight loss, sarcopenia and systemic inflammation on oncological outcomes are well known and observable in curative and palliative settings [10, 11, 12, 13, 14, 15].

However, there is little information on whether anti‐cachexia interventions are more effective in mild or more severe activity of the cachexia syndrome [16, 17].

Kumar et al. allude to the potential effect of combining or sequencing anti‐cachexia and anti‐cancer strategies [9]. In our analysis of 57 trials [2], there were only combined treatments, but none in sequence. However, when considering a treatment sequence, it may both appear suboptimal to delay oncological therapy while waiting for an anti‐cachexia effect or to delay cachexia treatment until completing the oncological intervention. Another option would be interspersing anti‐cachexia and anti‐cancer activities.

Symptoms of cachexia are of prime relevance to the diseased subjects, and drug approval agencies increasingly require documentation of improvement in patient‐reported outcomes (PRO) [18]. In fact, PRO are covered extensively in our endpoint series by Hjermstad et al. [3]. Due to a lack of respective data, analysing interactions between oncological endpoints and PROs was not possible in our review [2] and thus needs to be positioned as an important goal for newly designed trials.

Finally, we agree that when considering treatment options for cancer cachexia, the socioeconomic background is of major importance on a local and especially on a global scale. We thus urge to aim for low‐cost and easily available but effective measures to improve nutritional and metabolic status. Comparing the 57 studies analysed, 49 were performed in high‐income countries, while only 2 were from lower middle and 6 from upper middle income countries [19]. Unfortunately, judging differential effectivity is impossible from these data given the heterogeneity in trial design and the lack of effectivity in 55 of the 57 trials.

In conclusion, future trials targeting anti‐cachexia interventions should be designed to (1) control for the degree of cachexia, interactions with anti‐cancer therapies and socioeconomic settings and (2) compare effects on oncological with those on patient‐reported outcomes.

## Conflicts of Interest

J.A. has received personal fees for consultancy from Danone and Pfizer. R.J.E.S. has received personal fees for consultancy from Artelo, Actimed, Faraday and Helsinn. B.J.A.L. has received personal fees for consultancy from Artelo, Actimed, Faraday, Kyona Kirin and Toray.
